# The Effect of Whey on Performance, Gut Health and Bone Morphology Parameters in Broiler Chicks

**DOI:** 10.3390/foods9050588

**Published:** 2020-05-05

**Authors:** Vasileios Tsiouris, Michael G. Kontominas, Giorgos Filioussis, Sofia Chalvatzi, Ilias Giannenas, Georgios Papadopoulos, Konstantinos Koutoulis, Paschalis Fortomaris, Ioanna Georgopoulou

**Affiliations:** 1 Unit of Avian Medicine, Clinic of Farm Animals, Aristotle University of Thessaloniki, Stavrou Voutyra 11, 54627 Thessaloniki, Greece; biltsiou@vet.auth.gr (V.T.); ioannag@vet.auth.gr (I.G.); 2 Laboratory of Food Chemistry, Department of Chemistry, School of Sciences, University of Ioannina, 45110 Ioannina, Greece; kontominas@gmail.com; 3 Laboratory of Microbiology and Infectious Diseases, Aristotle University of Thessaloniki, Stavrou Voutyra 11, 54627 Thessaloniki, Greece; georgefilious@gmail.com; 4 Laboratory of Animal Husbandry, Aristotle University of Thessaloniki, Stavrou Voutyra 11, 54627 Thessaloniki, Greece; schalvat@vet.auth.gr (S.C.); geopaps@vet.auth.gr (G.P.); fortomap@vet.auth.gr (P.F.); 5 Laboratory of Nutrition, School of Veterinary Medicine, Faculty of Health Sciences, Aristotle University of Thessaloniki, Stavrou Voutyra 11, 54627 Thessaloniki, Greece; 6 Department of Poultry Diseases, Faculty of Veterinary Science, University of Thessaly, Trikalon 224, 43100 Karditsa, Greece; koutoulis@yahoo.gr

**Keywords:** broiler chicken, whey, bone strength, performance, caecal microbiota

## Abstract

Whey is a highly nutritious byproduct of the cheese industry that can be used effectively in the animal feed industry. However, the use of whey in poultry diets is limited by its high lactose and mineral contents. The objective of the present study was to evaluate the effect of different concentrations of whey in poultry diets on the performance, intestinal microbiota and physico-chemical parameters of the intestinal ecosystem, as well as on the bone morphology and its strength in broiler chicks. One hundred and twenty-eight, day-old, male broiler chicks were randomly allocated into four treatment groups of 32 chicks each. The treatment groups were: group A, which served as negative control and groups B, C and D, supplemented with 1, 2 and 5% of dietary whey, respectively. Performance of the groups was evaluated throughout the experiment. Following necropsies, the gastrointestinal tract from each bird was removed, divided into its anatomical parts and intestinal samples were taken for microbiological analysis and for pH and viscosity measurement as well. Tibiotarsus was also collected for morphometric analysis and strength evaluation. The statistical analysis of the experimental data revealed that the dietary supplementation of 1 and 2% of whey improved significantly (*p* ≤ 0.05) the body weight, while the addition of 5% of whey reduced significantly (*p* ≤ 0.05) the body weight. Furthermore, the addition of 1, 2 and 5% of dietary whey increased significantly (*p* ≤ 0.05) the pH of jejunum digesta and reduced significantly (*p* ≤ 0.05) the pH of caecum digesta compared to the control group. The addition of 1 and 2% of whey reduced significantly (*p* ≤ 0.05) the viscosity in the jejunum and ileum digesta, compared to the addition of 5% of whey which reduced significantly (*p* ≤ 0.05) the viscosity in jejunum digesta but increased significantly (*p* ≤ 0.05) the viscosity in ileum digesta. Moreover, the addition of 1, 2 and 5% of dietary whey increased significantly (*p* ≤ 0.05) the caecal counts of Lactobacillus spp. and Lactococcus lactis, while the addition of 5% of whey reduced significantly (*p* ≤ 0.05) the tibiotarsus length. It can be concluded that the addition of low quantities of whey up to 2% promoted the performance and gut health of birds, while the addition of higher quantities of whey at the level of 5% had a detrimental effect on the performance and tibiotarsus length.

## 1. Introduction

Whey is the valuable by-product of the cheesemaking process resulting after the production of cheese, whey cheese or casein from milk. It represents 90% of the volume and 50% of the solids of milk and it is characterized by its high concentrations of water, lactose and sodium [1,2]. 

Whey disposal is problematic, due to its high organic matter content and resultant high biological oxygen demand. Large scale commercial cheese plants usually process whey to recover milk solids, which are used in the food or feed industry. However, remote or small cheese plants find it difficult to process whey due to either low quantities of whey produced or due to high capital investment of whey processing facilities required. Hence, whey quantities disposed to the environment, such as rivers and water resources, could result in environmental pollution and nutrient losses [2,3,4].

Whey can no longer be regarded as waste material under a revaluation prism of environmentally friendly practices and circular economy, which aims at eliminating waste and the continual use of resources. It has already been used as a dietary ingredient in the feed industry i.e., in piglet diets. Thus, a solution to its problematic disposal is proposed, providing additional economic benefits for the animal industry [4]. However, dry whey has not been extensively applied in the poultry industry yet, which could potentially utilize large quantities of whey. The use of whey in poultry diets is limited mainly because of its high lactose and sodium contents. Poultry species are not mammals and they have not made any evolutionary adaption to produce lactase, the enzyme necessary to hydrolyze the milk sugar lactose into glucose and galactose [2,5]. 

Besides its nutrition properties, whey has been used to control intestinal pathogens, such as *Salmonella* spp., *Campylobacter* spp. and *Clostridium perfringens* in broiler chicks [6,7,8]. A plausible mechanism is that lactose acts as a substrate for fermentation by intestinal lactic-acid producing bacteria and thus the pH of the intestinal digesta is also affected [9]. Furthermore, lactose enhances the intestinal absorption of calcium and phosphorus, which could affect the strength and morphology of bones [5,10,11]. 

Whey has been evaluated in the literature as poultry feed ingredient with controversial outcomes [2,11,12,13]. This was mainly attributed to the use of products of variable composition, with different lactose and protein concentrations utilized either in dried or liquid form. Therefore, the objective of the current study was to evaluate the effect of different dietary inclusion levels of whey in poultry diets on the performance, intestinal microbiota and physico-chemical parameters of intestinal digesta, as well as on the strength and morphology of tibiotarsus bones in broiler chicks. Second investigation was whether wheat can be substituted by whey since both contain similar crude protein content; in that case the main comparison is that wheat starch is substituted in the diet by whey lactose.

## 2. Material and Methods

### 2.1. Birds and Housing 

One hundred and twenty-eight, 1-day old, Ross^®^ 308, male broiler chicks were obtained from a local commercial hatchery and were randomly allocated into four groups of 32 chicks each with 4 replicates per treatment group. All birds were wing tagged and placed in pens with a deep litter of wood shavings, which were previously sterilized in an autoclave at 121 °C for 20 min (Cyclomotic control, EA605A). 

All groups were kept in the specially designed experimental facilities of the Unit of Avian Medicine, School of Veterinary Medicine, Faculty of Health Sciences, Aristotle University of Thessaloniki, EL54BIO03, where the temperature, relative humidity and lighting were controlled, following the recommendations of the breeder. One replicate from each group was kept in the same cage divided into four parts, while each group was replicated in four separate rooms. Temperature and humidity were monitored in each room at bird level using a temperature-humidity record system (HOBO UX100-003 Temperature/Relative Humidity data logger, Onset Computer Corporation, 470 MacArthur Blvd., Bourne, MA 02532, USA). Detailed daily temperature and humidity values are provided in Supplementary Table S1.

### 2.2. Experimental Diets

To meet the nutrient requirements of the broiler chicks during the experimental period, three complete different basal diets were formulated (starter 1–13 d, grower 14–23 d and finisher 24–37 d) for the starter, growing and finishing periods, respectively. The addition of whey powder was done at the expense of wheat in grower and finisher rations. Feed formulation and chemical analysis of feed rations are presented in Table 1, whereas the chemical and microbiological analysis of the commercial whey powder product is presented in Table 2. No antibiotic growth promoters, anticoccidials, organic acids or phytobiotics were used. Feed and drinking water were offered to birds *ad libitum* throughout the experiment.

### 2.3. Experimental Design

The study received the approval of Veterinary Directorate of Central Macedonia and was conducted in compliance with the Ethical Committee of Aristotle University guidelines. The groups used in this study were: group **A**, which served as a control and groups **B**, **C** and **D**, supplemented with 1, 2 and 5% of dietary whey respectively. Four birds per replicate per sampling day were randomly removed on the 23rd and 37th day of age. The birds were euthanized by exposure to a rising concentration of carbon dioxide in an air-tight container and were subjected to necropsy. The gastrointestinal tract was removed immediately and divided into its anatomical parts.

### 2.4. Performance

The body weight (BW) of birds was measured individually on the 1st, 13th, 23rd and 37th day of age, while the feed consumption (FC) and feed conversion ratio (FCR) were calculated for the periods of the 1st–13th day, 14th–23rd day, 24th–37th day and cumulatively. Mortality was recorded daily.

#### 2.4.1. pH

The digesta of the duodenum, jejunum, ileum and caecum from each bird were immediately collected after euthanasia in separate tubes and vortexed to obtain a homogenous content from each anatomical part of intestine per bird. The pH of the duodenum, jejunum, ileum and caecum from each bird was measured using a digital pH-meter (pH 315i, WTW Wissenschaftlich- TechnischeWerkstätten, Weilheim, Germany). Two readings were taken from each sample and the average was presented.

#### 2.4.2. Viscosity

The viscosity of intestinal digesta was determined as described by Tsiouris et al. [14]. The homogenous content of the jejunum and the ileum from each bird, was filled in separate Eppendorf tubes (1.5 mL). The tubes were centrifuged at 3000× *g* for 45 min to separate the feed particles from the liquid phase. Supernatants (0.5 mL) from each tube were taken and the viscosity was measured in a Brookfield DV-II+ PRO Digital Viscometer (Brookfield Engineering Laboratories, Stoughton, MA, USA). Two readings were taken from each sample and the average was expressed in centipoise (cP).

#### 2.4.3. Determination of Caecal Microbiota

The content of the cecum was squeezed out into a sterile 15-mL container. One gram of the content was transferred in 9 mL sterile PBS and homogenized. Furthermore, serial tenfold dilutions ranking from 10^3^ to 10^7^ were prepared. The MRS agar (Merck 110660, Darmstadt, Germany) was used for the enumeration of *Lactobacillus* spp. and the plates were incubated anaerobically at 37 °C for 48 h for the calculation of LAB. *Lactococcus* spp. viable counts were enumerated on M17 agar plates incubated at 30 °C under anaerobic conditions for 3 days. *Clostridium perfringens* was enumerated on TSC agar (Merck 111972, Darmstadt, Germany), incubated at 37 °C for 24 h anaerobically. For the enumeration of total anaerobic bacteria Plate Count Agar (PCA) was used and anaerobic incubation at 37 °C was carried out for 48 h. Anaerobiosis was achieved by applying the inoculated plates in jar. The anaerobic environment was generated by the use of Anaerocult^®^ A (Merck 1.13829) and was confirmed by the use of Anaerotest^®^ (Merck 1.15112). Finally, *Escherichia coli* was enumerated on MC agar, incubated at 37 °C for 24–48 h aerobically. *Lactobacilli* were confirmed to the genus level according to Kymario et al. [15] while *E. coli* and *C. perfringens* were identified to the species level according to Quin et al. [16] and Engberg et al. [17]. The identification of isolated *Lactococus* spp. on M17 agar was achieved by API test that reveals *Lactococuc lactis species* according to Roy et al. [18]. Results were expressed as base-10 logarithm colony-forming units per gram of caecal digesta.

#### 2.4.4. Bone Morphometric Analysis and Strength

At the age of 37th day, 4 birds per replicate per treatment group were weighed and sacrificed via carbon dioxide inhalation. The right tibiotarsus was excised, placed in individual plastic bags, and stored at −20 °C. Before analysis, bones were thawed overnight and cleaned of all tissue without boiling. Parameters such as weight, length and midpoint width were measured before bone breaking strength (breaking force divided by bone weight and expressed as kilograms per gram) were determined using a three-point bending test (TA.HD. plus Texture analyzer, Stable Microsystems Ltd., Surrey, UK). Bone weight was measured by Electronic balance model Navigator TM (N2B110 OHAUS Corporation, Nanikon, Switzerland) and bone length and midpoint width by Electronical digital caliper (EMC, Shenzhen, China).

### 2.5. Statistical Analysis

Both parametric and nonparametric statistical methods were applied for the statistical evaluation of the experimental results. For accessing the assumptions of normality and stability of variances, data were transformed to log_10_ or sqrt. In the case of normality and variance’s homogeneity, a one-way analysis of variance (OΝΕ-WAΥ ANOVA) was performed, to evaluate possible significant effects of treatment on the performance, the gross lesions in the intestine, as well as on the pH of the duodenum, jejunum, ileum and caeca and the viscosity of the jejunum and ileum and on the caecal microbiota. Differences between mean values of specific treatments were evaluated using Duncan’s new multiple range test. Whether assumptions regarding either variability or the form of the populations’ distribution were seriously violated, with or without transformed data, the Kruskal-Wallis nonparametric test was applied to evaluate treatment dependent differences, while differences between mean values of specific treatments were evaluated using the nonparametric Wilcoxon rank-sum test (Mann-Whitney U-test). All analyses were conducted using the statistical software program SPSS for Windows (v. 15.0). Significance was declared at *p* ≤ 0.05. Back-transformed mean values are reported in the results.

## 3. Results and Discussion

Whey is a highly nutritious byproduct of the cheese industry, which may substantially affect performance, bone morphology, microbiota and physico-chemical parameters in the intestinal tract of poultry. Lactose, the main ingredient of whey, acts as a prebiotic since it cannot be digested by the birds, but it is fermented by the intestinal microbiota [2,11,12,13,19].

The feed conversion ratio and average daily feed consumption data are presented in Table 3. They were collected and calculated on a pen basis. It can be concluded that whey inclusion levels in the diet higher than 2% may negatively affect birds’ performance. Therefore, the effect of whey at various concentrations in poultry diets should be further evaluated regarding lactose, sodium, chloride and protein contents. 

The body weight in all experimental groups was not significantly different (*p* > 0.05) at 1st, 13th and 23rd day of age (Table 3). However, the addition of 1–2% of whey in poultry diets increased significantly (*p* ≤ 0.05) the final body weight on day 37, whereas the addition of 5% whey decreased significantly (*p* ≤ 0.05) the final body weight, compared to control group. Moreover, the addition of 1–2% of whey in poultry diets reduced the feed conversion ratio for the periods of a 23rd–37th day of age and 1st–37th day compared to the control diet, whereas addition of 5% of whey in poultry diets significantly (*p* ≤ 0.05) deteriorated the cumulative feed conversion ratio. The addition of whey did not affect the mortality rate of birds in any group since no dead birds were recorded at any group.

The positive effect of low quantities of whey up to 4% in poultry performance has been reported in many studies [12,19,20,21]. In another recent study, it was shown that growth of chickens was improved by dietary incorporation of either 6% dry whey powder or 8% dry whey concentrate because of a higher mineral availability, increased feed intake and modulation of caecal microbiota communities [11]. Whey is an excellent source of nutrients, providing high quality protein, peptides, amino acids, lactose, minerals, vitamins and varying quantities of lipids [3,4].

In addition, whey contains a wide range of bioactive components, such as β-lactoglobulin, α-lactalbumin, immunoglobulins and peptides that pose a range of biological and physiological effects, such the regulation of skeletal muscle protein synthesis, reduction of oxidative stress and regulation of cellular processes [1,3]. In particular, whey proteins contain a high level of branched-chain amino acids (leucine, isoleucine, and valine), essential amino acids (cysteine) and peptides, which are considered an excellent amino acid source for birds [3,4]. These proteins have a higher biological value compared to the main protein source in poultry feed, soybean meal, which could further promote the performance of broiler chicks [11].

According to the Gülşen et al. [21] and Ocejo et al. [22], the improvement in the performance after whey dietary supplementation could be attributed to its effect on the intestinal integrity and morphology. In particular, the addition of whey resulted in a significantly (*p* ≤ 0.05) greater villus height. The larger villus area means that the absorptive surface of the intestinal tract is increased, leading to a more efficient nutrient utilization and, subsequently to enhanced performance of broiler chicks [23].

On the other hand, the addition of high concentrations of whey reduced the bodyweight of birds, which could be attributed to the lactose intolerance and/or increased osmolarity, due to its sugar and mineral content. In the current study, the maximum concentration of whey was 5% of a whey product containing 73% of lactose. This is equivalent to a dietary inclusion level of 3.65% of lactose, just above the bird’s lactose tolerance level of 3.5% [2,7]. 

The effect of whey, at various concentrations in the poultry diets, on the pH and viscosity values of the digesta of broiler chicks on the intestinal parts is displayed in Table 4. The addition of 5% whey increased significantly (*p* ≤ 0.05) the pH of duodenum digesta compared to other experimental groups, while the addition of 1, 2 and 5% whey increased significantly (*p* ≤ 0.05) the pH of jejunum digesta and reduced significantly (*p* ≤ 0.05) the pH of caecum digesta compared to the control group A. Ileal viscosity values were reduced significantly (*p* ≤ 0.05) by whey addition at all inclusion levels, however, this was noticed in jejunum at the very low whey inclusion level of 1%. Viscosity is related with main absorption sites, as low viscosity values allow higher absorption rates [8]. For that reason measurement of viscosity is less meaningful at caecal digesta or the very first part of duodenum.

The pH of the intestinal digesta depends on the feed composition and subsequently on the fermentation activity of intestinal microbiota [24,25]. The effect of whey on the pH of intestinal digesta has been observed in many studies [13,26]. The most plausible explanation for the effect of whey on the pH of the intestinal digesta is the lactose fermentation by the intestinal microbiota of the lacto- and bifido- bacteria. Lactose fermentation leads to the production of lactic acid and subsequently to the reduction of the pH value of intestinal digesta [13]. 

In our study, it was noticed that dietary increase of whey was accompanied with a slight increase in pH values in proximal parts of the intestine, especially in duodenum and jejunum in a dose-dependent manner, whereas pH values in the ileal digesta was remained unaffected. However, the dietary inclusion of whey affected caecal content that remains longer time under stable fermentation process, by presenting considerably lower pH values. Caecum is the main site for bacterial fermentation in chickens due to its special habitat [27]. Bacteria metabolize soluble nondigestible carbohydrates (sNDCs) into short chain fatty acids (SCFAs) and lactate which consequently lowers the pH [28]. Reduced pH may inhibit the growth of acid sensitive bacteria such as members of the family Enterobacteriaceae [13,26,28,29].

Viscosity, a physico-chemical property of the intestinal digesta, is associated with the feed materials, mainly the undigested part, and the digestive chyme [30,31]. It has been calculated that birds’ lactose tolerance is about 3.5%. If higher concentrations of lactose are used in the birds’ feed, then the excess of lactose remains undigested and osmotic diarrhoea may be observed leading to growth retardation [2,23]. The results of the present study confirmed the results of the previously published studies since the addition of 5% of whey increased significantly (*p* ≤ 0.05) the viscosity in ileum digesta and reduced the bodyweight of birds. On the other hand, the addition of low quantities of whey (1–2%) reduced significantly (*p* ≤ 0.05) the viscosity in ileum digesta and increased the bodyweight of birds. Low quantities of whey, although not absorbed by the digestive system of the birds, may act as a prebiotic and are used as a substrate by the intestinal microbiota, promoting gut health and performance [13]. In this study, evaluation of different carbohydrate substrates such as starch and pentozans of different grains or lactose at levels 30 g/kg or inulin supplemented at a concentration of 20 g/kg of feed, were tested.

Increased viscosity in the gastrointestinal tract of poultry, through addition of cereals containing high amounts of soluble non-starch polysaccharides (NSPs), has been shown to negatively interfere with digestion and absorption of nutrients and consequently reduce their performance [32]. Although many studies have investigated the anti-nutritive effect of increased gastrointestinal contents’ viscosity [28].

Poultry diets with high soluble NSP increase intestinal viscosity that hampers the nutrient digestibility and have deleterious effects on the bird’s health and performance. New generation carbohydrases may help in digestion of a broad range of dietary fibers with reduction of intestinal viscosity and competition between host and microbiota for SCFA in the small intestine and improve digestibility of nutrients. This enzyme activity may also reduce the loads of pathogenic microbes and improve intestinal health [33].

According to the data presented in Table 5, dietary whey had a significant effect (*p* ≤ 0.05) on the caecal microbiota of broiler chicks. In particular, the addition of 1, 2 and 5% of whey increased significantly (*p* ≤ 0.05) the caecal counts of *Lactobacillus* spp. compared to the control group, which confirms its effect on the pH of intestinal digesta. Furthermore, the addition of 5% whey increased significantly (*p* ≤ 0.05) the total anaerobic counts in the caeca compared to control group possibly due to the available quantity of lactose or lactic acid on sight. As mentioned above, lactose in chicks’ acts as a prebiotic, since it cannot be hydrolyzed or absorbed in the intestinal tract of chicks and is fermented by the intestinal microbiota stimulating the growth of beneficial caecal bacteria [11,13]. 

It is generally acknowledged that normal intestinal microflora is associated with increased numbers of *Lactobacilli* and decreased numbers of *E. coli* that can partly explain improvement in healthy status especially of young animals. In opposite cases, increased numbers of *E. coli* are associated with high incidence of diarrhoea in young animals causing mortality and growth retardation [23]. In our study we found that counts of *Lactococcus lactis* were increased. Recently, an excellent review of Markowiak and Śliżewska [34] explained in detail that lactic acid that is produced after bacterial fermentation of sugars, is partly dissociated, while the undissociated form passes through lipid cell membranes and by dissociating within the cell, it acidifies cell contents and inhibits the growth of pathogenic microorganisms, including putrefactive bacteria, Gram-negative bacteria, and also some moulds. Also, it was reported that lactic acid can be further fermented into acetic acid, which neutralizes electrochemical cell potential and can decrease the growth of putrefactive bacteria, including those of the genera *Clostridium* spp. and *Salmonella* spp. Bacteria of the genera *Lactobacillus* spp., *Enterococcus faecium, Lactococcus lactis* and *Streptococcus thernophilus* are the main producers of bacteriocins [18,34]. However, in the current study, the bacteriocin content in the gut was not evaluated. 

Elevated intestinal coliform and *E. coli* counts are generally associated with adverse health effects. These bacteria are often contrasted with *Lactobacillus* spp. [35]. On the contrary, the outcomes in some novel studies hinted a relation between higher intestinal *E. coli/Enterobacteriaceae* load and improved performance [36], meaning that the interrelationships of intestinal microbiota need further elucidation. It is generally accepted that indigenous *Lactobacillus* spp. are considered beneficial bacteria as they positively contribute to microbial balance and gut health through competitive exclusion and through the production of lactic acid [37]. In the study of Molnar et al. [30] diets with high sNDC content did not increase caecal *Lactobacillus* spp. numbers. Instead, sNDC diets resulted in a microbial shift towards a higher caecal coliform load relative to the control group. This notice of higher caecal coliform numbers was associated with enhanced intestinal functions such as lower caecal pH or higher butyrate concentration; pointing out an augmented bacterial fermentation in the tested sNDC groups due to higher substrate availability. 

Another similar study showed that high viscosity is also associated with higher incidence of necrotic enteritis (NE) [38]. Mortality due to NE and intestinal lesions of randomly selected birds were associated with substantially higher median *Clostridium perfringens* (CP) counts than those found in bird groups without such lesions. Those findings suggested that median counts above one million per gram predicted a high probability of concurrent NE-specific gut lesions in at least 10% of randomly selected, killed birds [38]. It is obvious that intestinal microbiota can play a major role mainly in the escalation of intestinal diseases. Very recently, it has been shown that coccidian diseases can modify the homeostasis of this dynamic ecosystem, negatively affect gut health and growth performance [39]. Although, the gastrointestinal ecosystem is well organized and very complicated that can be regarded resistant to changes, even in presence of a coccidian infection [39] the severity of clinical coccidiosis in individual chickens was quantified by microbial changes associated with different lesion scores. The coccidian infection, despite the fact that diversity of taxa within the caecal microbiome remained largely stable, it paused major changes in the abundance of microbial taxa. The major changes were detected in birds displaying severe caecal pathology; taxa belonging to the order Enterobacteriaceae were increased, while taxa from Bacillales and Lactobacillales were decreased. Notable were the profiles in birds that remained asymptomatic (lesion score 0), with taxa belonging to the genera Bacteroides to be decreased, whereas Lactobacillus be increased. Many differential taxa from the order Clostridiales were identified supporting the view that caecal microbiome dysbiosis was associated with Eimeria infection [39]. 

In Table 6, the effect of diet supplementation with whey is presented on bone morphology and breaking strength of tibiotarsus. 

The weight and diameter of the tibiotarsus, as well as the ratio breaking strength to bone weight, were not significantly different (*p* > 0.05) among experimental groups. But the addition of 5% of whey reduced significantly (*p* ≤ 0.05) the tibiotarsus length compared to the control group. The results of our study are in accordance with the results of the study of Scott [40] who observed that the addition of 5% whey resulted in an increase of the occurrence of enlarged hocks in young turkeys, while the addition of delactosed whey, resulted in the absence of enlarged hocks. Although the underlying mechanism has not been elucidated, it can be hypothesised that increased viscosity reduces the absorption of minerals and fat-soluble vitamins having a deleterious effect on the occurrence of enlarged hocks [41]. It was an interesting finding that dietary addition of whey at levels of 1 and 2% did not adversely affect tibiotarsus bone weight and breaking strength. The importance of retaining bone health is clear, as intensive genetic selection for performance in modern broilers has increased growth rate but at the same time has compromised skeletal development and integrity [42]. Skeletal problems, particularly those affecting leg bones are associated with chronic pain in broilers [43] and high prevalence of lameness causing substantial financial losses due limitations in access to feed [42,44]. Several factors such as genetics, gender, endocrinal hormones, growth and aging, nutrition and health status, toxins and antinutrient compounds and physical loading can either directly or interactively affect bone strength in poultry [45]. Therefore, healthy bone development should be at first sight of examination on research trials with new dietary components [46].

The current manuscript addresses a value-added potential for an agricultural waste such as cheese whey that offers potential poultry feed additive benefits, while reducing the negative effects of such a food waste and its accompanied environmental hazards. In addition, it has been successfully used to control intestinal pathogens, such as *Salmonella* spp., *Campylobacter* spp. and *Clostridium perfringens* in broiler chicks. The novelty of the current research is that whey may be used in low inclusion levels with positive effects on the growth performance, physico-chemical properties of intestinal digesta and the intestinal microbiota, while the high inclusion levels of whey may have deteriorated effects on the performance and bone characteristics. Therefore, the poultry industry could be an alternative reservoir absorbing large quantities of whey and minimizing environmental pollution. However, further studies are needed in order to investigate possible whey interaction or synergy with other feed additives, such as probiotics, prebiotics and organic acids, as well as its effect on the gut immunity.

## Figures and Tables

**Table 1:** Composition and chemical analysis of the basal poultry diets used in the experiment.

Ingredients (g/kg)	Starter				Grower				Finisher			
	A	B	C	D	A	B	C	D	A	B	C	D
	0% whey	1% whey	2% whey	5% whey	0% whey	1% whey	2% whey	5% whey	0% whey	1% whey	2% whey	5% whey
Maize	290.0	290.0	290.0	290.0	150.0	150.0	150.0	150.0	150.0	150.0	150.0	150.0
Wheat	301.0	291.0	281.0	251.0	486.0	476.0	466.0	436.0	577.0	567.0	557.0	527.0
Soybean meal	335.0	335.0	335.0	335.0	286.0	286.0	286.0	286.0	203.0	203.0	203.0	203.0
Palm oil	8.00	8.00	8.00	8.00	20.0	20.0	20.0	20.0	24.0	24.0	24.0	24.0
Soybean oil	22.0	22.0	22.0	22.0	20.0	20.0	20.0	20.0	10.0	10.0	10.0	10.0
Whey	0.0	10.0	20.0	50.0	0.0	10.0	20.0	50.0	0.0	10.0	20.0	50.0
Calcium carbonate	15.2	15.2	15.2	15.2	15.4	15.4	15.4	15.4	15.4	15.4	15.4	15.4
Xylanase	0.10	0.10	0.10	0.10	0.10	0.10	0.10	0.10	0.10	0.10	0.10	0.10
Phytase	0.05	0.05	0.05	0.05	0.05	0.05	0.05	0.05	0.05	0.05	0.05	0.05
Vitamin & mineral premix *	28.0	28.0	28.0	28.0	22.0	22.0	22.0	22.0	20.0	20.0	20.0	20.0
**TOTAL**	1000	1000	1000	1000	1000	1000	1000	1000	1000	1000	1000	1000
	**Calculated analysis**											
Moisture (%)	11.45	11.45	11.41	11.38	11.17	11.17	11.14	11.11	11.16	11.16	10.96	10.86
Crude protein (%)	22.50	22.50	22.50	22.50	21.30	21.30	21.30	21.30	18.30	18.30	18.30	18.30
Crude fat (%)	4.80	4.80	4.60	4.40	5.85	5.85	5.65	5.45	6.30	6.30	6.15	6.05
Crude fibre (%)	3.46	3.46	3.42	3.39	3.25	3.25	3.21	3.15	2.99	2.99	2.89	2.79
Starch (%)	34.8	34.3	33.8	32.8	36.1	35.6	35.1	34.1	39.6	39.1	38.6	37.6
Ash (%)	6.03	6.03	6.13	6.23	5.79	5.79	5.89	5.99	5.51	5.51	5.61	5.71
Metabolized energy (Kcal/kg)	3000	3000	3000	3000	3060	3060	3060	3060	3185	3185	3185	3185

* Supplying per kg feed: 13,000 IU vitamin A, 4000 IU vitamin D3, 40 mg vitamin E, 9 mg vitamin K, 3 mg thiamin, 7 mg riboflavin, 6 mg pyridoxine, 0.035 mg vitamin B12, 40 mg niacin, 13 mg pantothenic acid, 1.5 mg folic acid, 0.13 biotin, 340 mg choline chloride, 55 mg Zn, 155 mg Mn, 20mg Fe, 12 mg Cu, 0.2 mg Co, 1 mg I, 0.2 mg Se.

**Table 2:** Chemical and microbiological analysis of commercial whey powder product.

**Chemical Analysis**	
Crude protein (%)	11.0
Lactose (%)	73.0
Fat (%)	1.1
Ash (%)	10.0
Humidity (%)	5.0
Calcium (g/Kg)	4.5
Phosphorus (g/Kg)	5.5
Sodium (g/Kg)	18.6
Potassium (g/Kg)	17.0
Magnesium (g/Kg)	1.3
Chlorine (g/Kg)	40.4
Metabolisable energy, Kcal/kg	2,750
**Microbiological analysis**	
Total microbes (cfu/g)	< 5.0 × 10^4^
*Enterobacteriaceae* (cfu/g)	< 10.0
Yeast & fungi (cfu/g)	< 10.0
*Staphylococcus aureus*	Absence, not detected
*Salmonella* spp.	Absence, not detected

**Table 3:** Effect of whey in various concentrations in poultry diets on the body weight (BW), feed conversion ratio (FCR) and average daily feed consumption (ADFC).

**AGE**	**Experimental Groups**					
	**A**	**B**	**C**	**D**		
	**0% whey**	**1% whey**	**2% whey**	**5% whey**		
	**BW (Kg)**				***SEM***	***p***
**1st day**	0.040	0.039	0.039	0.039	0.002	NS
**13th day**	0.435	0.429	0.431	0.432	0.031	NS
**23rd day**	1.168	1.165	1.180	1.137	0.092	NS
**37th day**	2.438 ^b^	2.586 ^a^	2.607 ^a^	2.239 ^c^	0.152	0.041
	**FCR**					
**1st–13th day**	1.03	1.04	1.04	1.04	0.006	NS
**14th–23rd day**	1.35 ^a,b^	1.39 ^b^	1.34 ^b^	1.42 ^a^	0.009	0.008
**24th–37th day**	1.96 ^a^	1.73 ^b^	1.93 ^a^	1.85 ^a,b^	0.014	<0.001
**1st–37th day**	1.74 ^a^	1.51 ^b^	1.60 ^b^	1.75 ^a^	0.017	<0.001
	**ADFC (Kg)**					
**13th–23rd day**	0.10	0.10	0.10	0.10	0.001	NS
**24th–37th day**	0.20	0.17	0.19	0.17	0.002	NS

^a, b^ Mean values in the same row with a different superscript differ significantly (*p* ≤ 0.05), NS = Non significant.

**Table 4:** Effect of whey in various concentrations in poultry diets on the pH and viscosity of intestinal digesta of broiler chicks.

	**Experimental Groups**					
	**A**	**B**	**C**	**D**		
	**0% whey**	**1% whey**	**2% whey**	**5% whey**	***SEM***	***p***
	**pH**					
**Duodenum**	5.96 ^b^	6.04 ^b^	6.03 ^b^	6.16 ^a^	0.02	0.018
**Jejunum**	5.19 ^b^	5.58 ^a^	5.62 ^a^	5.54 ^a^	0.01	0.017
**Ileum**	5.40	5.54	5.59	5.58	0.01	NS
**Caecum**	6.53 ^a^	6.10 ^b^	6.05 ^b^	6.01 ^b^	0.02	0.018
	**Viscosity (Centipoise)**					
**Jejunum**	2.23 ^a^	1.51 ^c^	1.88 ^b^	1.82 ^b^	0.01	0.015
**Ileum**	2.42 ^a^	2.14 ^c^	2.62 ^ab^	2.85 ^b^	0.01	0.014

^a, b, c^ Mean values in the same row with a different superscript differ significantly (*p* ≤ 0.05). NS = Non significant.

**Table 5:** Effect of whey in various concentrations in poultry diets on cecal bacterial counts (log cfu/g).

	Experimental Groups					
	A	B	C	D		
	0% whey	1% whey	2% whey	5% whey	*SEM*	*p*
***Lactobacillus* spp.**	5.16 ^b^	6.15 ^a^	6.24 ^b^	6.34 ^a^	0.02	0.015
***Lactococcus lactis***	1.08 ^b^	1.36 ^a^	1.26 ^a^	1.38 ^a^	0.04	0.019
***C. perfringens***	4.50	4.32	4.32	4.49	0.11	NS
***E. coli***	1.24	1.17	1.31	1.29	0.09	NS
**Total anaerobic bacteria**	5.52 ^b^	6.77 ^a^	6.52 ^b^	6.72 ^a^	0.062	0.029

^a, b^ Mean values in the same row with a different superscript differ significantly (*p* ≤ 0.05). NS = Non significant.

**Table 6:** Effect of whey in various concentrations in poultry diets on the morphology and breaking strength of tibiotarsus.

Parameter	Experimental Groups					
	A	B	C	D		
	0% whey	1% whey	2% whey	5% whey	*SEM*	*p*
**Bone weight (g)**	14.04	13.68	13.47	12.60	0.52	NS
**Bone length (mm)**	77.78 ^a^	76.50 ^ab^	76.93 ^ab^	74.88 ^b^	0.71	0.009
**Bone diameter (mm)**	9.95	9.93	9.68	9.53	0.59	NS
**Breaking strength/**						
**Bone weight (kg/g)**	2.78	2.90	2.90	3.00	0.06	NS

^a,b^ Mean values in the same row with a different superscript differ significantly (*p* ≤ 0.05). NS = Non significant.

## Supplementary Materials

The following are available online at https://www.mdpi.com/2304-8158/9/5/588/s1.
